# Characterization of bacterial-type phospho*enol*pyruvate carboxylase expressed in male gametophyte of higher plants

**DOI:** 10.1186/1471-2229-10-200

**Published:** 2010-09-14

**Authors:** Tomoko Igawa, Masayuki Fujiwara, Ichiro Tanaka, Yoichiro Fukao, Yuki Yanagawa

**Affiliations:** 1 The Plant Science Education Unit, The Graduate School of Biological Sciences, Nara Institute of Science and Technology, 8916-5 Takayama-cho, Ikoma, Nara 630-0101, Japan; 2 Graduate School of Nanobioscience, Yokohama City University, 22-2 Seto, Kanazawa-ku, Yokohama 236-0027, Japan; 3 National Institute of Crop Science, National Agriculture and Food Research Organization, 2-1-18 Kannondai, Tsukuba, Ibaraki 305-8518, Japan; 4 Current Address: Initiative Research Program, Advanced Science Institute, RIKEN, 2-1 Hirosawa, Wako, Saitama 351-0198, Japan

## Abstract

**Background:**

Phospho*enol*pyruvate carboxylase (PEPC) is a critical enzyme catalyzing the β-carboxylation of phospho*enol*pyruvate (PEP) to oxaloacetate, a tricarboxylic acid (TCA) cycle intermediate. PEPC typically exists as a Class-1 PEPC homotetramer composed of plant-type PEPC (PTPC) polypeptides, and two of the subunits were reported to be monoubiquitinated in germinating castor oil seeds. By the large-scale purification of ubiquitin (Ub)-related proteins from lily anther, two types of PEPCs, bacterial-type PEPC (BTPC) and plant-type PEPC (PTPC), were identified in our study as candidate Ub-related proteins. Until now, there has been no information about the properties of the PEPCs expressed in male reproductive tissues of higher plants.

**Results:**

Expression analyses showed that lily BTPC (LlBTPC) and *Arabidopsis *BTPC (AtBTPC) were significantly expressed in pollen. The fusion protein AtBTPC-Venus localized in the cytoplasm of the vegetative cell (VC). Both LlBTPC and AtBTPC expression initiated after the last mitosis before pollen germination. Lily PTPC (LlPTPC) and monoubiquitinated LlPTPC (Ub-LlPTPC) remained at constant levels during pollen development. In late bicellular pollen of lily, LlBTPC forms a hetero-octameric Class-2 PEPC complex with LlPTPC to express PEPC activity.

**Conclusion:**

Our results suggest that an LlBTPC:Ub-LlPTPC:LlPTPC complex is formed in the VC cytoplasm during late pollen development. Both LlBTPC and AtBTPC expression patterns are similar to the patterns of the appearance of storage organelles during pollen development in lily and *Arabidopsis*, respectively. Therefore, BTPC is thought to accelerate the metabolic flow for the synthesis of storage substances during pollen maturation. Our study provides the first characterization of BTPC in pollen, the male gametophyte of higher plants.

## Background

Phospho*enol*pyruvate carboxylase (PEPC, EC4.1.1.31) catalyzes the irreversible β-carboxylation of phospho*enol*pyruvate (PEP) to yield oxaloacetate and inorganic phosphate (Additional file 1). PEPC exists widely in plants, algae, and bacteria, but not in animals or fungi [1]. In plants, PEPC acts as an allosteric enzyme and is phosphorylated by PEPC protein kinase [1-3]. Active PEPC commonly consists of a plant-type PEPC (PTPC) homotetramer, and is typically inhibited by L-malate and aspartic acid and activated by glucose-6-phosphate (Glc-6-P). PEPC has been extensively studied in C4 and CAM photosynthesis, because it is a critical enzyme catalyzing the initial reaction of atmospheric CO_2 _fixation [1]. It also plays pivotal metabolic roles in nonphotosynthetic and C3 photosynthetic cells, particularly in the anaplerotic replenishment of the TCA cycle intermediates consumed during lipid synthesis [4], biosynthesis, and nitrogen assimilation [5]. The genomic analysis of the PEPC of *Arabidopsis *and rice first revealed that higher plants contain a small PEPC family containing two types of PEPC, PTPC and bacterial-type PEPC (BTPC) [6]. BTPC resembles the bacterial PEPC rather than the common plant PEPC in terms of its gene structure and the absence of an N-terminal seryl-phosphorylation domain, a hallmark of PTPC (Additional file 2). Recent studies have indicated that BTPC in developing castor oil seeds (COS) interacts with PTPC to form a heterooctameric complex with PEPC activity [7,8].

Recently, PTPC was reported to be a ubiquitinated protein in *Arabidopsis *[9,10] and a monoubiquitinated protein in germinated COS [11]. Ubiquitination is one of the major protein modifications that occur in all eukaryotic cells, and is critical for the regulation of various cellular functions, such as DNA damage repair, endocytosis, endosomal sorting, and signal transduction, in addition to proteolysis by the 26 S proteasome [12]. We previously established a method for the purification and identification of Ub-related proteins (ubiquitinated proteins and their associated proteins) in *Arabidopsis *seedlings [13]. Applying this method, we purified and identified PTPC and BTPC from the lily anther as candidate Ub-related proteins (Additional files 3 and 4).

**Figure 1:**
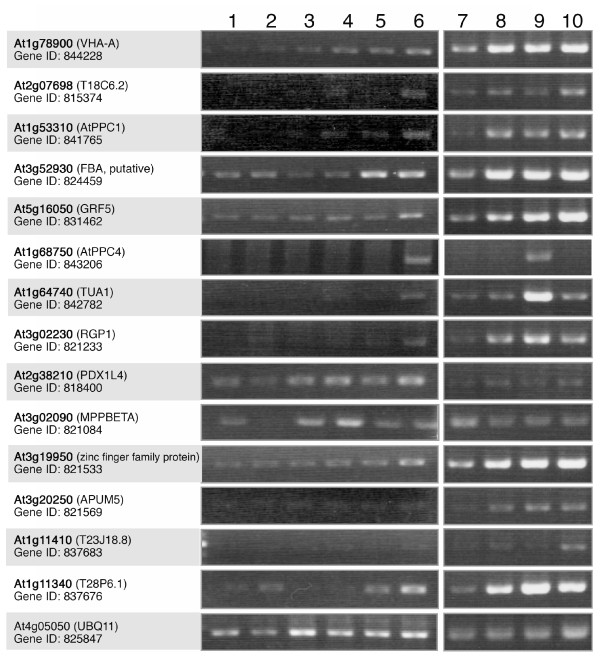
**Expression pattern of the putative orthologous genes in various *Arabidopsis *organs determined by RT-PCR analysis**. AGI code, gene name, and gene ID for each *Arabidopsis *orthologue are represented by bold letters, shown in parentheses, and found on the 2nd line, respectively. *Ubiquitin 11 *(UBQ11, At4g05050) was used as the standard. 1, seedlings; 2, roots; 3, rosette leaves; 4, lateral leaves; 5, stems; 6, flowers; 7, sepals; 8, petals; 9, stamens; 10, pistils.

As far as we know, no previous study has focused on the PEPC expressed in plant reproductive tissues. Because AtBTPC showed significant expression in stamen (Figure 1) and COS BTPC is reported to interact with PTPC [7,8], we focused here on BTPC and PTPC in male reproductive tissues. In this study, we analyzed the expression, localization, and interaction of BTPC and PTPC in lily and *Arabidopsis*. Our results suggest that BTPC forms a complex with PTPC and monoubiquitinated PTPC (Ub-PTPC) to accelerate the accumulation of storage substances during pollen maturation.

## Results

### BTPC and PTPC are identified as Ub-related proteins in lily anther, and AtBTPC shows stamen-specific expression

With the large-scale purification of Ub-related proteins from the lily anther, 13 proteins were identified as candidate Ub-related proteins by liquid chromatography-tandem mass spectrometry (LC-MS/MS). The limited number of proteins identified is probably attributable to the small available database of lily proteins. Nineteen distinct orthologous *Arabidopsis *genes were determined based on the database (Additional file 4). Five candidate proteins (vacuolar H^+^-ATPase subunit, F1 ATPase, fructose-bisphosphate aldolase-like protein, pyridoxine biosynthesis protein-like, and metalloendopeptidase) have been reported in the *Arabidopsis *pollen proteomes [14,15]. Moreover, two orthologous *Arabidopsis *genes (TAIR: At1g78900 and At3g02230) have been reported to be essential for pollen development [16,17].

To determine the expression patterns of the identified candidate Ub-related proteins, the mRNA expression of putative *Arabidopsis *orthologues were examined because of the limited genomic information available for the lily. Fourteen orthologous genes were selected for RT-PCR analysis in various vegetative tissues, and all of the genes investigated were expressed in flowers. The expression of each gene was examined in the flower organs. All the genes were expressed in the stamen (Figure 1). Among them, the transcripts of α-tubulin 1 (TUA1; TAIR At1g64740) and AtBTPC (*Atppc4*; TAIR At1g68750) showed almost stamen-specific expression. TUA1 is already known to be expressed specifically in pollen [18,19]. Therefore, we focused our attention on BTPC. We found that one of the PTPC orthologous genes, *Atppc1 *(TAIR: At1g53310), is also expressed in stamens (Figure 1). PTPC has also been identified as a Ub-related protein and they are reported to interact in COS [7,8], so both types of PEPC were investigated in the following analysis.

**Figure 2:**
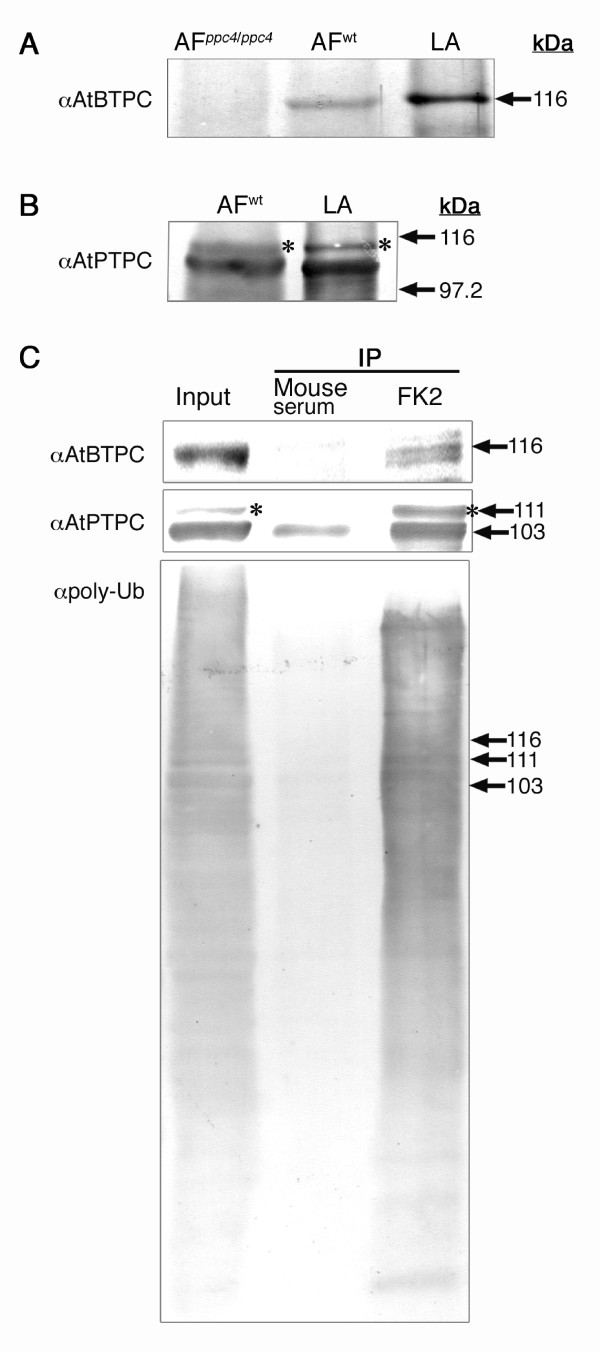
**Specificities of the antibodies produced and immunoprecipitation**. (A) An anti-AtBTPC antibody recognized an AtBTPC band of the expected size (ca. 116 kDa) in flower bud proteins from wild-type *Arabidopsis *(AF^wt^) and an LlBTPC band of equivalent size in the lily anther (LA) proteins. No AtBTPC band was detected in the flower bud proteins from the *atppc4 *mutant plants (AF^*ppc4/ppc4*^). (B) The anti-AtPTPC antibody recognized an AtPTPC band of the expected size (ca. 110 kDa) in the flower bud proteins from wild-type *Arabidopsis *(AF^wt^) and an LlBTPC band of equivalent size in the lily anther (LA) proteins. Note that the anti-AtPTPC antibody recognized AtPPC1, AtPPC2, and AtPPC3 because of the high homology of their amino acid sequences (see Additional file 2). (C) Immunoprecipitation with FK2 of LlBTPC (upper) and LlPTPC (middle) from lily anthers containing late bicellular pollen. Mouse serum was used as the control. Note that FK2 immunoprecipitated greater amounts of LlPTPC (lower band) than did mouse serum. The upper bands detected with anti-AtPTPC antibody (asterisks) likely represent monoubiquitinated LlPTPC (Ub-LlPTPC). The proteins precipitated with FK2 were immunoblotted with a polyclonal antibody directed against Ub (αpoly-Ub), which detects both ubiquitinated protein and free Ub (bottom), confirming that FK2 precipitates Ub-related proteins.

To verify that BTPC and PTPC are Ub-related proteins, lily anther proteins were immunoprecipitated with anti-Ub antibody (FK2; Nippon Bio-Test Laboratories, Tokyo, Japan), which selectively recognizes the Ub moiety but not free Ub [20]. As expected, both bands representing LlBTPC and LlPTPC, of the expected sizes (see Figure 2A and 2B for the specificities of the antibodies), were co-immunoprecipitated with FK2, indicating that they are the Ub-related proteins in the lily anther (Figure 2C). Another band, larger than the expected size of LlPTPC, was also detected with the anti-AtPTPC antibody (asterisks in Figure 2B and 2C). The 'larger' anti-AtPTPC antibody immunoreactive band was approximately 8 kDa larger than the 'smaller' immunoreactive band and showed similar mobility to the monoubiquitinated PTPC (Ub-PTPC) band in germinated COS [11]. The larger bands were also co-immunoprecipitated with FK2, but not with mouse serum (Figure 2C), indicating that the larger bands were probably Ub-LlPTPCs.

Large-scale purification enabled the identification of truncated sizes of BTPC and PTPC (Additional files 3 and 4), indicating that the *in vitro *proteolytic cleavage of the two proteins occurred during protein extraction and purification (these procedures require approximately 10 hours). In contrast, immunoprecipitated Ub-related proteins contained the expected sizes of BTPC and PTPC (Figure 2C). The size discrepancies of purified BTPC and PTPC between these two immuno-purification experiments may be due to the different purification periods because *in vitro *COS BTPC proteolysis in an inappropriate buffer was also observed when the incubation period was extended [7]. It seemed like that the anti-AtBTPC antibody detected two bands (Figure 2C), implying the truncation of BTPC by proteolysis or post-translational modification (e.g., monoubiquitination or phosphorylation), although these possibilities need to be confirmed in future studies.

### LlBTPC is specifically expressed in pollen after pollen mitosis I (PMI) and forms a complex with LIPTPC

**Figure 3:**
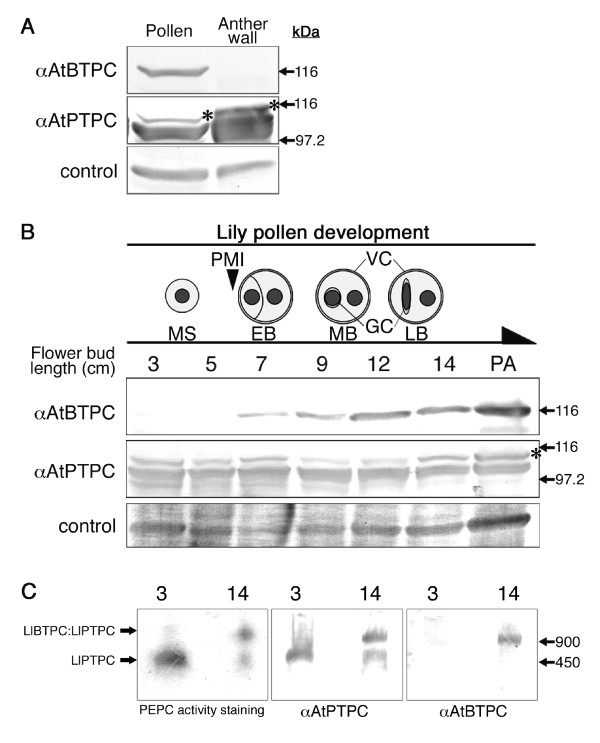
**Expression analysis of lily PEPCs in male tissues and developing pollen**. (A) Expression of LlBTPC and LlPTPC in anther tissues. Proteins (10 μg) from the pollen and anther wall of 14 cm flower buds were treated with anti-AtBTPC (upper) or anti-AtPTPC (middle) antibody. Non-specific reactive bands were used as loading control (bottom). The upper bands detected with anti-AtPTPC antibody (asterisk) represent Ub-LlPTPC. (B) Expression patterns of LlBTPC and LlPTPC in developing microspores and pollen. Top diagram illustrates lily pollen development, each stage of which corresponds to the length of the flower bud (cm). Protein extracts from lily pollen (10 μg) were subjected to immunoblot analysis with anti-AtBTPC (upper) or anti-AtPTPC (middle) antibody. The upper bands detected with anti-AtPTPC antibody (asterisk) represent Ub-LlPTPC. Non-specific reactive bands were used as loading control (bottom). PMI, pollen mitosis I; MS, microspore; EB, early bicellular pollen; MB, mid bicellular pollen; LB, late bicellular pollen; GC, generative cell; VC, vegetative cell; PA, post anthesis. (C) Nondenaturing PAGE analysis of microspore and late bicellular pollen proteins (7 μg). The numbers in each panel represent the length of the flower bud (cm) containing the microspores (3) or late bicellular pollen (14). In-gel staining for PEPC activity (left), and immunoblot analysis with anti-AtPTPC (middle) or anti-AtBTPC (right) antibody were performed.

To clarify the tissue specificity of LlBTPC and LlPTPC within the lily anther, the anthers of 14 cm flower buds were dissected into the anther wall and pollen, and an immunoblot analysis was performed with anti-AtBTPC and anti-AtPTPC antibodies. As shown in Figure 3A, LlBTPC specifically existed in the pollen, whereas LlPTPC was detected in both pollen and the anther wall.

The expression of both PEPCs was examined in pollen at various developmental stages. During pollen development, an asymmetric division of the haploid microspores, called PMI, produces two differently fated cells, a larger VC and a smaller generative cell (GC). The GC, which is enclosed within the VC, undergoes a second mitotic division called "pollen mitosis II" (PMII), giving rise to two sperm cells (SCs) before fertilization [21,22]. PMII in the lily pollen occurs after pollen germination, so the mature pollen is bicellular at anthesis. The lily pollen developmental stages are distinguishable based on the flower bud length [23] (Figure 3B, top diagram). Flower buds of 3-5 cm contain microspores, and PMI is completed by the 7 cm flower bud stage [24]. The GC morphology changes from round to spindle-like shape in the 12-14 cm stages [25], and the pollen reaches maturity at anthesis. Immunoblot analysis of LlBTPC during pollen development demonstrated that LlBTPC accumulation starts after PMI, and the amount increases to the 12 cm flower bud stage, and is sustained at this level until after anthesis (Figure 3B). LlPTPC and Ub-LlPTPC remained at constant levels during pollen development.

To investigate the interaction between the LlBTPC and LlPTPC proteins and their PEPC activity in pollen, the lily pollen proteins were separated by nondenaturing polyacrylamide gel electrophoresis (PAGE), followed by in-gel staining for PEPC activity and immunoblot analysis (Figure 3C). In the microspores at the 3 cm flower bud stage, the band showing PEPC activity only reacted with the anti-AtPTPC antibody. In pollen from the 14 cm flower buds, two PEPC active bands were detected and the smaller band only reacted with the anti-AtPTPC antibody, whereas the larger band reacted with both the anti-AtPTPC and anti-AtBTPC antibodies. The molecular masses of the smaller and larger bands were approximately 450 and 900 kDa, respectively. This indicates that LlBTPC interacts with LlPTPC, forming an active PEPC complex in late bicellular pollen.

### *AtBTPC *promoter is preferentially active in pollen

**Figure 4:**
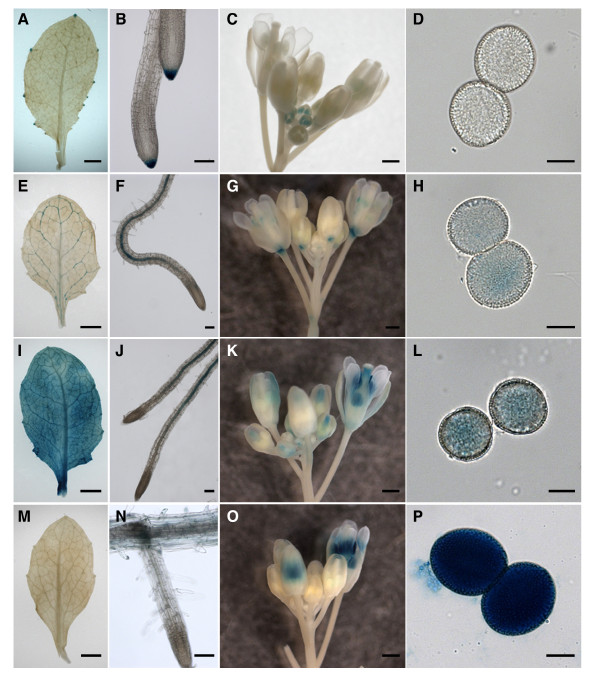
**Activities of *Atppc1-4 *promoters in different organs**. Histochemical localization of GUS reporter gene expression in rosette leaf (A, E, I, M), root (B, F, J, N), flower buds (C, G, K, O), and mature pollen (D, H, L, P) driven by the *Atppc1 *(A-D), *Atppc2 *(E-H), *Atppc3 *(I-L), or *Atppc4 *(M-P) promoter. Bars = 0.5 mm in A, C, E, G, I, K, M, and O; 50 μm in B and N; 100 μm in F and J; and 10 μm in D, H, L, and P.

Because the lily is refractory to molecular analysis, the expression patterns of *Arabidopsis *PEPC orthologues were analyzed of detail in plant tissues. The *Arabidopsis *PEPC gene family contains three PTPC genes (*Atppc1 *[TAIR: At1g53310], *Atppc2 *[TAIR: At2g42600], and *Atppc3 *[TAIR: At3g14940]) and one BTPC gene (*Atppc4 *[TAIR: At1g68750]; Additional file 2). Transgenic plants were produced in which the β-glucuronidase (GUS) reporter gene was placed under the control of the promoter of each *Atppc *gene. To roughly compare the expression levels of these genes, all the transgenic lines were incubated with the substrate for the same time. As expected, the *AtBTPC *gene promoter *ProAtppc4 *showed significant activity in mature pollen (Figure 4P). Strong GUS staining was detected in the stamens in the late flower bud stage, whereas it was never observed in the younger flower buds (Figure 4O). Faint activity was observed in the root cortices of the same transgenic lines (Figure 4N), but no signal was observed in the leaves (Figure 4M), even with longer incubation. Transgenic plants expressing green fluorescent protein (GFP) under the control of *ProAtppc4 *showed specific GFP signal in the pollen within the stamens (data not shown), indicating that AtBTPC is expressed specifically in pollen. Conversely, weaker GUS staining was observed in the mature pollen in all transgenic lines when the GUS reporter gene was driven by the *AtPTPC *gene promoters (*ProAtppc1*-*3*). Among the *AtPTPC*s, *ProAtppc3 *activity seemed to be strongest in the stamen and pollen in the late flower bud stage (Figure 4C-D, G-H, and 4K-L). *ProAtppc1 *activity was negligible in mature pollen (Figure 4D), whereas GUS staining was observed in the stamens of the younger flower buds (Figure 4C). All the *AtPTPC *gene promoters were active in all the somatic tissues investigated here, but with different expression patterns (Figure 4A-C, E-G, and 4I-K).

### AtBTPC localizes in vegetative cell cytoplasm and shows limited expression during pollen development

**Figure 5:**
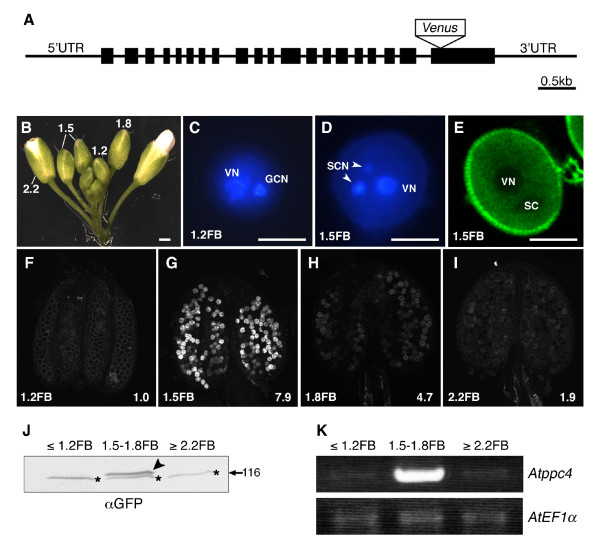
**Protein and gene expression of AtBTPC**. (A) Schematic view of the gene structure of genomic *Atppc4 *(At1g68750) used for expression analysis. Black boxes represent exons. The *Venus *gene was inserted at the *Bst*1107I site in the last exon. (B) *Arabidopsis *flower buds. The numbers indicate the flower bud length (mm). Scale bar = 0.5 mm. (C, D) DAPI staining of pollen in 1.2 mm flower bud (1.2FB; C) and in 1.5 mm flower bud (1.5FB; D). VN, vegetative cell nucleus; GCN, generative cell nucleus; SCN, sperm cell nucleus. Scale bar = 10 μm. (E) CLSM image of Venus fluorescence in pollen at stage 1.5FB. The blank regions correspond to VN and SC. Scale bar = 10 μm. (F-I) CLSM images of Venus fluorescence in pollen within an anther at stages 1.2FB-2.2FB. Hemizygous T3 plants were used for the analysis. Each number in the bottom right corner represents the relative fluorescence intensity. Note that the relative fluorescence intensity of the pollen at 1.2FB is given as 1.0, because there was no fluorescent signal in the transgenic pollen at this stage. (J) Immunoblot analysis with anti-GFP antibody of flower buds from homozygous T3 plants. Flower buds were analyzed at stages before 1.2FB (≤ 1.2FB) at 1.5FB-1.8FB, and after 2.2FB (≥ 2.2FB). The arrowhead indicates a band of AtPPC4-Venus protein. The lower bands with star marks in all lanes are nonspecific bands. (K) RT-PCR of *Atppc4 *in flower buds at different stages from wild-type plants. The stages of the flower buds are the same as in (J). *Arabidopsis *elongation factor 1α(*AtEF1α*, TAIR: At1g07920) was used as the standard.

To analyze the localization and the expression pattern of AtBTPC in the pollen during pollen development, "fluorescent tagging of full-length proteins technology" was applied, because this strategy was available to monitor the expression patterns and subcellular localization of *Arabidopsis *gene products in planta [26]. The transgenic plants were produced by introducing the native genomic *Atppc4 *gene, including the 5' and 3' untranslated regions (UTRs), coupled to *Venus *[27], which encodes a variant of yellow fluorescent protein, with low sensitivity to pH (Figure 5A). Unlike lily pollen, *Arabidopsis *pollen undergoes PMII before pollen germination, so the mature pollen is tricellular at anthesis. The stages of pollen development were distinguishable based on the flower bud length (Figure 5B). A notable fluorescent signal was observed in pollen at the 1.5 mm flower bud stage (1.5FB; Figure 5B, E, and 5G). 4', 6-Diamidino-2-phenylindole (DAPI) staining indicated that pollen at 1.5FB was tricellular (Figure 5D), whereas pollen preceding the 1.2 mm flower bud stage (1.2FB; Figure 5B) was bicellular (Figure 5C). Confocal laser scanning microscopic (CLSM) analysis of the pollen at 1.5FB showed that AtBTPC-Venus localized in the VC cytoplasm, whereas there was no signal in the VC nuclei or SCs (Figure 5E).

The levels of AtBTPC-Venus expression during pollen development were estimated by analyzing the fluorescent signal intensities (Figure 5F-I). Whereas no fluorescent signal was observed in the pollen at 1.2FB (Figure 5F), the signal with the most intense fluorescence appeared in the pollen at 1.5FB (Figure 5G). The intensity then decreased sharply in the later developmental stages (Figure 5H and 5I).

Immunoblot analysis of AtBTPC-Venus using anti-GFP antibody showed that the AtBTPC-Venus protein accumulated in the pollen at 1.5-1.8FB, supporting the results of the CLSM analysis (Figure 5J). To confirm the mRNA expression of endogenous AtBTPC in pollen, RT-PCR of full-length *Atppc4 *was performed. As expected, the *Atppc4 *transcripts accumulated strongly in the pollen at 1.5-1.8FB, similar to the AtBTPC-Venus protein (Figure 5K).

## Discussion

RT-PCR and promoter-GUS analyses of *Arabidopsis *PEPC genes revealed that AtBTPC was also preferentially expressed in stamen (Figure 1), particularly in pollen (Figure 4P), similar to lily, whereas AtPTPCs (AtPPC1-3) were expressed more strongly in somatic tissues. Unlike a previous study [6], however, the *Atppc1 *expression shown by our RT-PCR analysis was negligible in somatic tissues (Figure 1). This may be due to the reaction conditions or the developmental stages of the investigated tissues, because transgenic plants with *ProAtppc1*-GUS showed GUS staining in confined regions of leaf, root, and flower (Figure 4A-C). The difference in the *ProAtppc3*-GUS expression pattern from the RT-PCR result reported by Sánchez et al. [6] may be due to the same reason as that stated above and/or differences in mRNA and protein stability. Analysis with a reporter protein fused to each *Arabidopsis *PEPC would provide detailed information of AtPEPC expression.

BTPC is significantly expressed in the pollen of both *Arabidopsis *and lily (Figures 3A and 4), but the initiation of the expression of each BTPC differs during pollen development (Figures 3B and 5). LIBTPC starts to accumulate after GC formation, after PMI, whereas AtBTPC expression starts immediately after SC formation, after PMII. Based on these findings, it seems that the initiation of BTPC expression in pollen does not depend on the mitosis type, but is triggered after the 'last mitosis' during pollen development, before germination. In both bicellular and tricellular pollen, the expansion of the pollen grain, organelle differentiation, and dehydration occur after the last mitosis for pollen maturation [28]. However, pollen dehydration does not seem to affect *Atppc4 *expression, because *Atppc4 *expression started after PMII, but was not sustained until anthesis (Figure 5), although it has been reported that *Atppc4 *expression is inducible by drought stress in root tissues [29].

We examined the AtBTPC-null mutant line, *atppc4 *(Figure 2A), but no abnormal characteristics were observed in the mutant pollen when analyzed by DAPI staining and ultrastructural observation, or in plant growth or fertility (data not shown). Therefore, plant BTPC may not be an essential factor for pollen development or plant survival, at least under our experimental growth conditions. Nevertheless, the expression of BTPC is significant in the pollen, suggesting that BTPC plays an important role in pollen development.

It is well known that PEPC is a PTPC homotetramer. In a previous study with COS, the presence of low- and high-molecular-mass PEPC isoforms was reported, which were designated Class-1 (PTPC homotetramer, 410 kDa) and Class-2 PEPCs (PTPC:BTPC heterooctamer, 910 kDa), respectively [7,30]. After nondenaturing PAGE in our study, the small active PEPC band only reacted with anti-AtPTPC antibody, whereas the large active PEPC band reacted with both anti-AtPTPC and anti-AtBTPC antibodies (Figure 3C), showing the same immunoreactivities as COS Class-1 and Class-2 PEPCs, respectively [7]. The small and large active PEPC bands in lily showed similar sizes to those of the COS Class-1 and Class-2 PEPCs, respectively. Therefore, the lily PEPCs in the microspores and the late bicellular pollen exist as complexes, and the small and large PEPC complexes probably corresponded to the COS Class-1 and Class-2 PEPCs, respectively. It has also been reported that PTPC is monoubiquitinated in COS, resulting in a Class-1 PEPC heterotetramer comprised of Ub-PTPC:PTPC [11]. In this work, FK2 immunoprecipitated Ub-LlPTPC from the lily anther containing late bicellular pollen in addition to the native forms of LlBTPC and LlPTPC (Figure 2C), suggesting that the native forms of LIBTPC and LlPTPC are the associated proteins of ubiquitinated-LlPTPC resulting in the identification by large-scale purification of Ub-related proteins from lily anther (Additional file 3). In addition, Ub-LlPTPC was present together with LlPTPC in the pollen throughout its development from microspore to mature pollen (Figure 3B). From these results, we infer that the Class-1 PEPC complex in the microspore comprises Ub-LlPTPC:LlPTPC and the Class-2 PEPC complex in the late bicellular pollen comprises LlBTPC:Ub-LlPTPC:LlPTPC.

**Figure 6:**
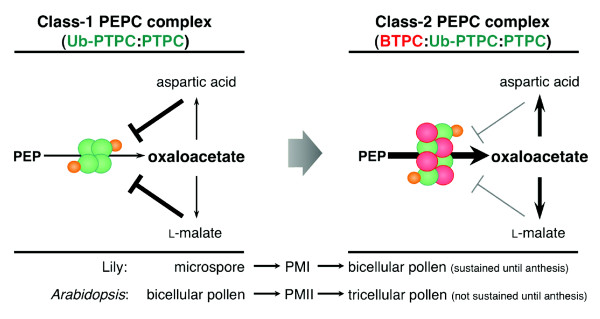
**Schematic model of the hypothetical role of BTPC in lily and *Arabidopsis *pollen development**. Before PMI in the lily microspore or before PMII in *Arabidopsis *pollen, PEPC occurs as a Class-1 heterotetrameric PEPC complex comprising PTPC (green circles) and Ub-PTPC (green circles with a smaller orange circle). Ub-PTPC:PTPC is allosterically inhibited by L-malate and aspartic acid metabolized from oxaloacetate. When BTPC (red circles) is expressed, PEPC becomes the Class-2 heterooctameric PEPC complex comprising BTPC:Ub-PTPC:PTPC and its sensitivity to inhibitors is reduced, whereas its sensitivity to activators is maintained. Accordingly, the metabolic flow from oxaloacetate to protein and lipid synthesis is enhanced during the process of pollen maturation.

PEPC is allosterically activated by Glc-6-P and inhibited by L-malate and aspartic acid metabolized from oxaloacetate (Additional file 1). Recently, it was reported that Class-2 PEPCs show much lower sensitivity to allosteric activators and inhibitors than do Class-1 PEPC homotetramers, suggesting that the interaction of PTPC and BTPC stabilize the metabolic flow under physiological conditions that would otherwise inhibit Class-1 PEPC [30-32]. It has also been reported that Class-1 PEPC heterotetramers of Ub-PTPC:PTPC are more sensitive to both activators and inhibitors than are Class-1 PEPC homotetramer of PTPC in COS [11]. These reported features and our results together suggest that expressed BTPC binds to the Ub-PTPC:PTPC complex, and that the resulting BTPC:Ub-PTPC:PTPC complex is even less sensitive to inhibitors, but maintains its higher sensitivity to activators relative to the sensitivity of Class-1 PEPC homotetramers. Consequently, it is speculated that the BTPC:Ub-PTPC:PTPC complex may stabilize and accelerate the metabolic flow required for lipid and protein synthesis in pollen (Figure 6). The increase in PEPC activity in developing COS endosperm was coincident with the onset of the most active phase of storage oil accumulation [33]. It has also been reported that the protein content of transgenic bean plants expressing a *Corynebacterium glutamicum *PEPC was elevated in their seeds by up to 50% [34]. In lily pollen, there is a marked increase in lipid bodies in the VC cytoplasm of the mature pollen compared with those in the pollen before PMI [35]. In *Arabidopsis *tricellular pollen, storage vacuoles accumulate after PMII and disappear from the mature pollen just before anthesis and at anthesis [36]. The patterns of the appearance of storage organelles in lily and *Arabidopsis *pollen almost coincide with the patterns of LlBTPC and AtBTPC expression, respectively, supporting our hypothesis that BTPC plays a role in the acceleration of metabolic flow to facilitate the synthesis of storage substances during late pollen development. Previous studies of Class-2 PEPCs have suggested that green alga and vascular plants have divergent PEPCs that serve to function in adaptation for survival in different environments [30-32,37]. It will be interesting to determine what triggers and suppresses the expression of BTPC during pollen development. Further investigation of Class-1 and Class-2 PEPCs is expected to provide more information of the metabolic pathway underlying the synthesis of storage substances in both bicellular and tricellular pollens during development.

## Conclusions

The large-scale purification of Ub-related proteins from the lily anther should advance research into the reproductive factors related to Ub-mediated protein modifications. In this study, BTPC was first found as a Ub-related protein in pollen.

LlBTPC and AtBTPC show significant expression in pollen and they are first expressed after the last mitosis before pollen germination. The duration of their expression almost coincides with the appearance of storage organelles during the maturation processes of both pollens. The presence of BTPC:Ub-PTPC:PTPC with PEPC activity during pollen maturation is also suggested in this study. It would be necessary to confirm the presence of the Ub-PTPC:PTPC and BTPC:Ub-PTPC:PTPC complexes with their kinetic properties in pollen in future studies. Furthermore, future tasks include clarification of the AtPTPC proteins (AtPPC1-3) involved in Class-1 and Class-2 PEPC formation during pollen development because AtPTPCs show different expression patterns (Figure 2), and investigation of their post-translational modifications, such as ubiquitination and phosphorylation. The results are expected to shed light on the role of PEPCs in pollen development.

BTPC is strongly expressed in the late stage of pollen development, but an AtBTPC-deficient mutant plant, *atppc4*, showed no abnormalities in pollen development, at least under our experimental growth conditions. PEPC itself is involved in the anaplerotic replenishment of intermediates of the TCA cycle, and for this reason, any obvious effects of BTPC deficiency may be difficult to identify. Alternatively, analysis under various physiological and growth conditions, such as varying temperatures, may identify the physiological function of BTPC in the reproductive process, because the thermal stability of Class-2 PEPC activity is relatively increased [32]. This is the first characterization of BTPC in pollen, the male gametophyte of higher plants. Our work provides new information for the study of the function of BTPC in plants.

## Methods

### Primers

Information on all primers used in this study is given in Additional file 5.

### Plant materials

Trumpet lily (*Lilium longiflorum *cv. Hinomoto) was grown in a greenhouse for immunoblot analysis and nondenaturing PAGE.

*Arabidopsis thaliana *(ecotype Columbia 0) was grown at 22°C under a 16/8 h light/dark cycle. A Salk line (SALK_144112; background Columbia) of *Atppc4 *was obtained from the Arabidopsis Biological Resource Center http://abrc.osu.edu/. The genotype of this line was confirmed by PCR using the gene-specific primer 'ppc4-SALK-For' and the T-DNA-specific primer 'LBc'. The T-DNA insertion site was verified by sequencing.

### Large-scale purification and identification of Ub-related proteins in lily anther

Anthers of 12-14 cm flower buds were collected from commercially obtained trumpet lily (*L. longiflorum*). Approximately 100 mg of total crude protein was obtained from 150 lily anthers. Protein extraction, purification with an anti-Ub antibody (FK2), in-gel digestion, and LC-MS/MS analysis were performed as described by Igawa et al. [13]. The MS/MS spectra were analyzed with the MASCOT search engine (MatrixScience, http://www.matrixscience.com) against an NCBI protein database of all plant species. The peptide sets were then summarized manually, and proteins encoding ubiquitin and proteasomal subunits were eliminated from the list.

### RT-PCR

Total RNA was prepared from various *Arabidopsis *organs using TRIzol^® ^Reagent (Life Technologies Japan, Ltd., Tokyo, Japan). cDNA was synthesized from l μg of total RNA with oligo(dT)_20 _primer using the Rever-Tra Ace-α kit (Toyobo Co., Ltd., Osaka, Japan).

### Antibodies, immunoblot analysis, and immunoprecipitation

To produce polyclonal anti-AtPTPC and anti-AtBTPC antibodies, the open reading frames encoding the 300 N-terminal amino acids of AtPPC1 (At1g53310) and AtPPC4 (At1g68750; Additional file 2) were amplified using the cDNA derived from seedlings and stamens, respectively. Primers 'PPC1-3AB-F' and 'PPC1-3AB-R' for *Atppc1 *and primers 'PPC4AB-F' and 'PPC4AB-R' for *Atppc4 *were used to add *Nde*I sites to the 5' termini and *Bam*HI sites to the 3' termini. The *Nde*I-*Bam*HI fragments of *Atppc1 *and *Atppc4 *were cloned into the expression vector pET28c (+) encoding 6×histidine at the N terminus. The plasmids generated were transformed into BL21 (DE3) cells. The purified histidine-tagged AtPPC1 and AtPPC4 were injected into rabbits as antigens. The purified antisera were designated anti-AtPTPC and anti-AtBTPC antibodies, respectively. Anti-GFP antibody (Living Colors^® ^A. v. Monoclonal Antibody [JL-8]; Takara Bio Inc., Otsu, Japan) and polyclonal anti-Ub antibody (Sigma-Aldrich, Missouri, USA) were purchased.

For the immunoblot analysis, lily pollen and *Arabidopsis *flower buds were ground in liquid N_2 _and homogenized with the buffer described by Gennidakis et al. [7]. The homogenates were centrifuged at 17,400 × g at 4°C for 15 min, and the supernatants were centrifuged again for 5 min to isolate the total protein extract. Immediately after the extraction, the protein extracts were treated with SDS sample buffer.

For immunoprecipitation with FK2 [20], the lily anther proteins were homogenized with the extraction buffer used for the large-scale purification of Ub-related proteins [13]. The homogenates were centrifuged at 17,400 × g at 4°C for 15 min. The lipid layer was removed and the supernatants were centrifuged again for 5 min to isolate the total protein extracts. The protein extracts were mixed with Protein A-Sepharose 6MB (Sigma-Aldrich) coupled to FK2, and then incubated for 3 h at 4°C. Immediately after the incubation, the immunoprecipitated proteins were treated with SDS sample buffer.

The molecular masses (kDa) in Figures 2A-B, 3A, B, 5J and Additional file 3 were marked according to the positions of marker proteins (Protein Marker Broad Range; New England BioLabs Inc., MA, USA). The molecular masses in Figure 2C and Additional file 4 were estimated by calculation based on the mobilities of marker proteins (Prestained Protein Marker Broad Range; Cell Signaling Technology, Inc., MA, USA).

### Nondenaturing PAGE and staining for PEPC activity

The lily pollen extracts were prepared with the buffer described by Uhrig et al. [8] with modifications: the omission of 2,2'-dipyridyl disulfide and the addition of 20 μL/mL Calbiochem's Plant Protease Inhibitor Cocktail (Merck KGaA, Darmstadt, Germany). Nondenaturing PAGE and staining for PEPC activity were as described by Law and Plaxton [38]. The approximate molecular masses (Figure 3C) were estimated by calculation based on the mobilities of marker proteins (NativeMark Unstained Protein Standard; Life Technologies Japan, Ltd.).

### Construction of plasmids and generation of Arabidopsis transgenic plants

*ProAtppc1*, *ProAtppc2*, and *ProAtppc3 *were amplified by genomic PCR and each PCR product was ligated into the pENTR™/D-TOPO vector (Life Technologies Japan, Ltd.), then transferred with the LR reaction (Gateway; Life Technologies Japan, Ltd.) to the destination vector pGWB203 [39] carrying the GUS reporter gene. *ProAtppc4 *was amplified with primers 'ppc4-promoterF' and 'ppc4-promoterR', which added a *Sal*I site to the 5' terminus and a *Bam*HI site to the 3' terminus. The *Sal*I-*Bam*HI fragment was cloned into pENTR™3C (Life Technologies Japan Ltd.), and then transferred into pGW203 by the LR reaction. The genomic region of *Atppc4 *from the translation start site to the 3' UTR (ca. 1 kb) was amplified with primers 'PPC4-1F' and 'PPC4-3UTR-R', which added a *Bam*HI site to the 5' terminus and a *Not*I site to the 3' terminus. The *Bam*HI-*Not*I fragment was cloned into pENTR™3C carrying *ProAtppc4*. *Venus*, amplified by PCR with primers 'VenusF2' and 'VenusR2', was inserted at the unique *Bst*1107I site in the resulting plasmid. Finally, the *ProAtppc4*::*genomic-Atppc4*+*venus *fragment was transferred to pGWB1 by the LR reaction. All the plasmids generated were transformed into *Agrobacterium tumefaciens *strain GV3101, which was used to infect wild-type *Arabidopsis *plants.

### GUS assays and DAPI staining

The GUS substrate solution contained 100 mM NaPO_4 _(pH 7.0), 10 mM EDTA, 2.5 mM K_3_(Fe[CN]_6_), 2.5 mM K_4_(Fe[CN]_6_), 0.1% Triton X-100, and 0.5 mg/L X-Gluc. The samples were incubated in the dark at 37°C for 1 h (pollen) or 5 h (leaf, root, and inflorescence). The pollen was stained with DAPI as described by Park et al. [40].

### Microscopy and image analysis

Specimens of GUS-assayed *Arabidopsis *inflorescences and DAPI-stained pollen were observed under a Stemi 2000-C (Carl Zeiss Co., Ltd., Tokyo, Japan) or Axioplan 2 microscope (Carl Zeiss Co., Ltd.), and photo images were captured with an AxioCam MRc using AxioVision software (Carl Zeiss Co., Ltd.).

Anthers at various flower bud stages, taken from hemizygous T3 plants expressing Atppc4-Venus, were observed with the CLSM FV1000 Inverted Confocal Microscope (excitation 458 nm and emission band path 530-630 nm; Olympus Corp., Tokyo, Japan) with FluoroView software (Olympus Corp.). The fluorescence intensities were quantified with the ImageJ software http://rsbweb.nih.gov/ij/. The average values for the wild-type and transgenic pollens were obtained from at least 20 pollen grains per anther at each developmental stage. The average values for transgenic pollen were then divided by the average value for the wild-type pollen to calculate the relative fluorescence intensities (pollen at 1.2FB showed no fluorescent signal, so the value was given as 1.0).

## Authors' contributions

TI and YY conceived the study and designed all the experiments. IT grew and prepared the lily samples. MF and YF performed the LC-MS/MS analysis. TI performed all other analyses and interpreted the experimental data. TI, IT, and YY participated in writing the manuscript. All the authors have read and approved the final manuscript.

## Supplementary Material

Additional file 1 **Simplified metabolic pathway diagram. This file shows a simplified metabolic pathway diagram for COS germinating seeds **[33]. PEPC catalyzes the irreversible reaction that produces oxaloacetate from PEP. PEPC activation leads to protein and lipid synthesis. PEPC activity is allosterically activated by Glc-6-P and inhibited by L-malate and aspartic acid. Click here for file

Additional file 2 **Amino acid alignment of the AtPEPC family**. The amino acid alignment of the AtPEPC family. Residues conserved among four or three paralogues are highlighted with black or gray, respectively. The serine residue marked with the red inverted triangle is a conserved phosphorylation site in PTPC. The red bars above and the blue bars below the alignment indicate the corresponding peptide sequences of AtPTPC and AtBTPC, respectively, which were detected in the lily anther with LC-MS/MS analysis. The amino acid sequences overlain with pale red and pale blue were used to produce the anti-AtPTPC and anti-AtBTPC antibodies, respectively. Click here for file

Additional file 3 **Immunopurified proteins with FK2 from lily anther**. Proteins immunoprecipitated with FK2 from lily anther were subjected to SDS-PAGE and stained with Flamingo™(Bio-Rad Laboratories, CA, USA). Clear bands (marked with lowercase letter) were excised and numbered smearing regions cut into 2-mm-long gel pieces were digested with trypsin for LC-MS/MS analysis. Click here for file

Additional file 4 **Table of Ub-related proteins identified from lily anther and the putative *Arabidopsis *orthologous genes**. This additional file contains a table of the Ub-related proteins identified in the lily anther and the putative *Arabidopsis *orthologous proteins. Candidate proteins with high reliability (MASCOT score > 40; *P *< 0.05) are listed. The gel position for each identified polypeptide corresponds to that in Additional file 3. The approximate size of each identified protein was estimated by calculation based on the mobilities of marker proteins (indicated on the left of the panel in Additional file 3). Each *Arabidopsis *orthologous protein was determined with a BLASTP search at the TAIR website http://www.arabidopsis.org/Blast/index.jsp based on the protein sequence indicated in the corresponding column. The expression of the genes marked with an asterisk was checked by RT-PCR (see Figure 1). Click here for file

Additional file 5 **Primers used in this study**. Primers used in this study. Click here for file
