# Supplementary figures and images for: Characterization of bacterial-type phosphoenolpyruvate carboxylase expressed in male gametophyte of higher plants
Source: BMC Plant Biol. 2010 Sep 14;10:200. doi: 10.1186/1471-2229-10-200 (PMC2956549; doi:10.1186/1471-2229-10-200)

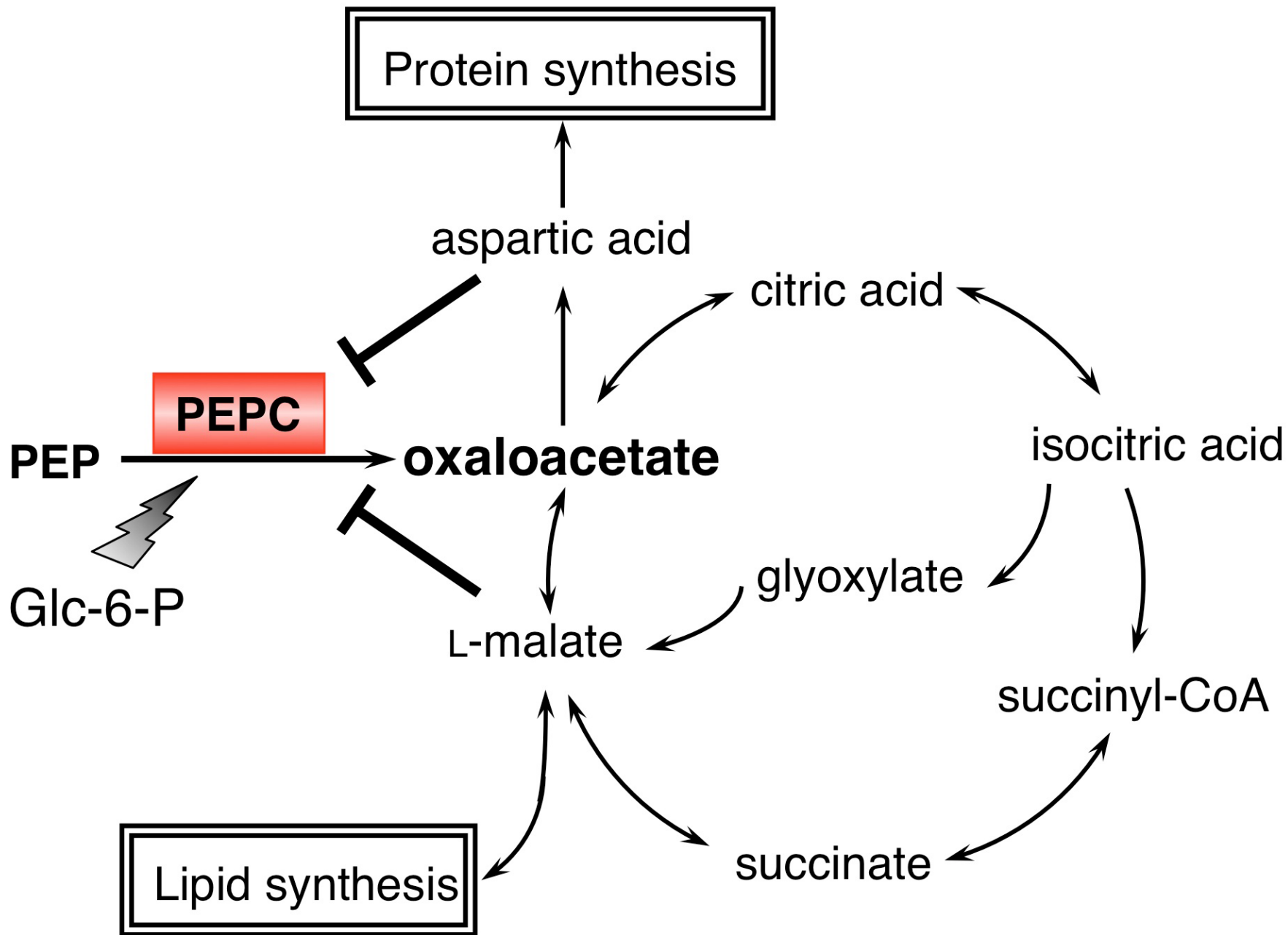

Supplement: Additional file 1 — Simplified metabolic pathway diagram. This file shows a simplified metabolic pathway diagram for COS germinating seeds [33]. PEPC catalyzes the irreversible reaction that produces oxaloacetate from PEP. PEPC activation leads to protein and lipid synthesis. PEPC activity is allosterically activated by Glc-6-P and inhibited by L-malate and aspartic acid. [file 1471-2229-10-200-S1.PDF]

# FK2

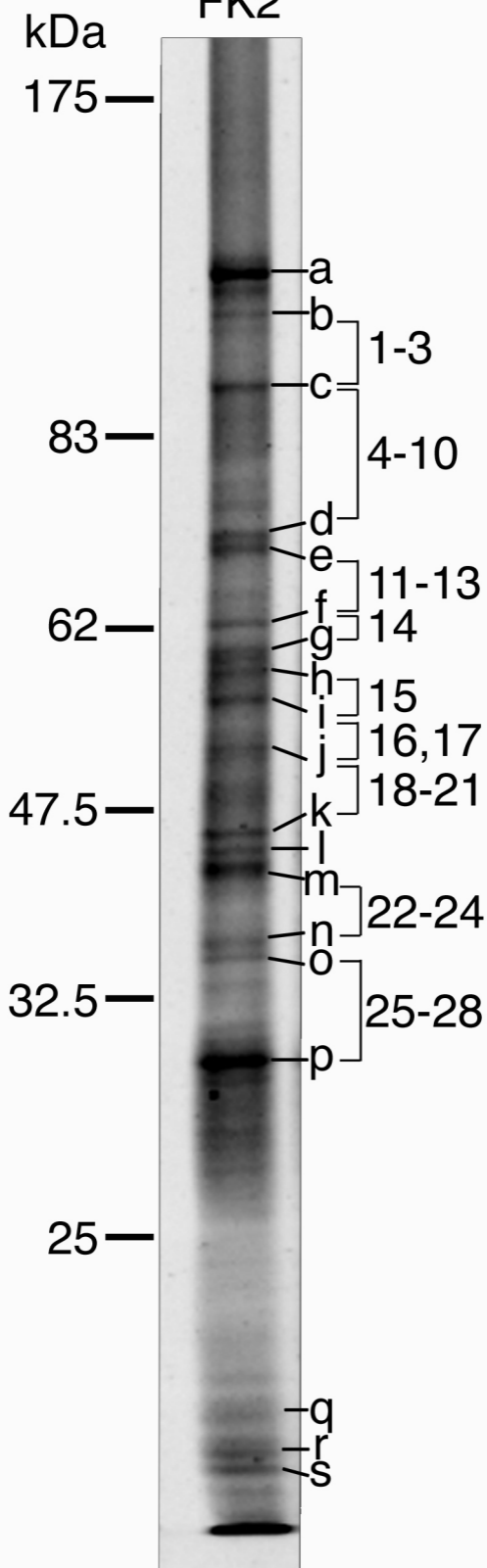

Supplement: Additional file 3 — Immunopurified proteins with FK2 from lily anther. Proteins immunoprecipitated with FK2 from lily anther were subjected to SDS-PAGE and stained with Flamingo™(Bio-Rad Laboratories, CA, USA). Clear bands (marked with lowercase letter) were excised and numbered smearing regions cut into 2-mm-long gel pieces were digested with trypsin for LC-MS/MS analysis. [file 1471-2229-10-200-S3.PDF]
