# Supplementary material for: Characterization of bacterial-type phosphoenolpyruvate carboxylase expressed in male gametophyte of higher plants
Source: BMC Plant Biol. 2010 Sep 14;10:200. doi: 10.1186/1471-2229-10-200 (PMC2956549; doi:10.1186/1471-2229-10-200)
Supplement: Additional file 2 — Amino acid alignment of the AtPEPC family. The amino acid alignment of the AtPEPC family. Residues conserved among four or three paralogues are highlighted with black or gray, respectively. The serine residue marked with the red inverted triangle is a conserved phosphorylation site in PTPC. The red bars above and the blue bars below the alignment indicate the corresponding peptide sequences of AtPTPC and AtBTPC, respectively, which were detected in the lily anther with LC-MS/MS analysis. The amino acid sequences overlain with pale red and pale blue were used to produce the anti-AtPTPC and anti-AtBTPC antibodies, respectively. [file 1471-2229-10-200-S2.PDF]

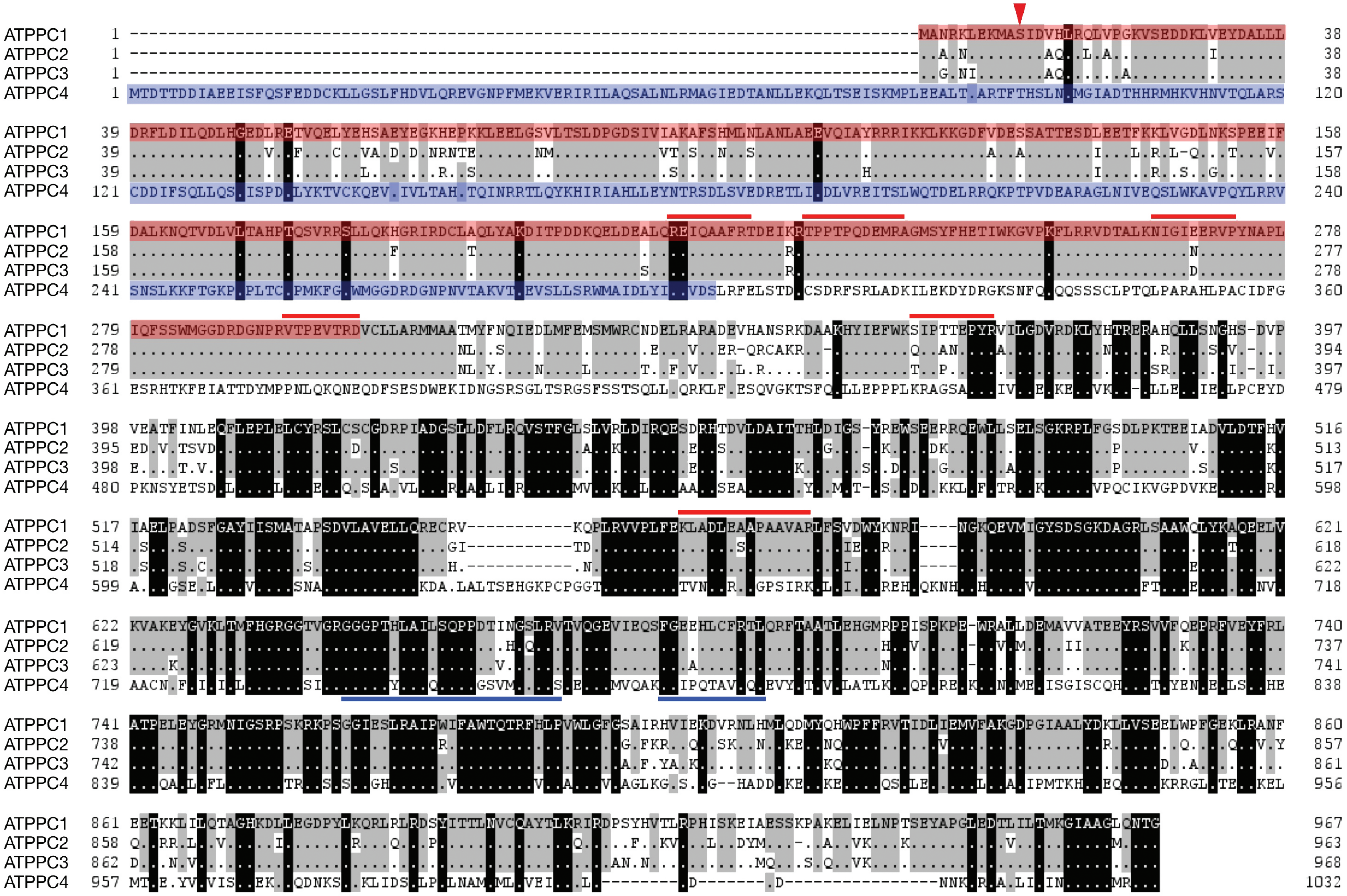
ATPPC1
ATPPC2
ATPPC3
ATPPC4
