# Supplementary material for: Characterization of bacterial-type phosphoenolpyruvate carboxylase expressed in male gametophyte of higher plants
Source: BMC Plant Biol. 2010 Sep 14;10:200. doi: 10.1186/1471-2229-10-200 (PMC2956549; doi:10.1186/1471-2229-10-200)
Supplement: Additional file 4 — Table of Ub-related proteins identified from lily anther and the putative Arabidopsis orthologous genes. This additional file contains a table of the Ub-related proteins identified in the lily anther and the putative Arabidopsis orthologous proteins. Candidate proteins with high reliability (MASCOT score > 40; P < 0.05) are listed. The gel position for each identified polypeptide corresponds to that in Additional file 3. The approximate size of each identified protein was estimated by calculation based on the mobilities of marker proteins (indicated on the left of the panel in Additional file 3). Each Arabidopsis orthologous protein was determined with a BLASTP search at the TAIR website http://www.arabidopsis.org/Blast/index.jsp based on the protein sequence indicated in the corresponding column. The expression of the genes marked with an asterisk was checked by RT-PCR (see Figure 1). [file 1471-2229-10-200-S4.PDF]

MASCOT score	Corresponding gel position†	Approx. size (kDa)	Peptide Set	Description	Protein sequences used for BLASTP @ TAIR		Putative *Arabidopsis* orthologue
					gi No.	Plant Species	
324	b	116	R.LASFYER.A	70kD vacuolar H^+-ATPase	gi|167560	*Daucus carota*	At1g78900*
	b, d	116, 83.5	R.SGDVYIPR.G				
	b, d	116, 83.5	R.TTLVANTSNMPVAAR.E				
	b	116	R.DMGYNVSMMADSTSR.W				
	c	105	K.TVISQALSK.Y				
	c	105	K.FEDPAEGEDVLVGK.F				
	d	83	K.DTVLELEFQGVK.K				
	d	83	R.EDDLNEIVQLVGK.D				
	d	83	R.LAEMPADSGYPAYLAAR.L				
283	g	68	R.VVSVGDGIAR.V	F1 ATPase	gi|12986	*Helianthus annuus*	At2g07698*
	g, 3	68, 105	K.AVDSLVPIGR.G				
	g	68	R.AAELTTLLESR.I				
	g	68	R.VVDALGVPIDGR.G				
	g, 3	68, 105	K.SVHEPMQTGLK.A				
185	26	32	R.EIQAAFR.T	Plant-type phospho*enol*pyruvate carboxylase	gi|23503558	*Lotus japonicus*	At1g53310*
	25	33	K.NIGINER.V		gi|18458	*Flaveria pringlei*	At2g42600
	25	33	R.VTPEVTR.D				At3g14940
	25	33	K.KISPGEPYR.I				
	26	32	K.LADLETAPAALAR.L				
	26	32	R.FVEYFR.L				
	o	35	R.LATPELEYGR.M				
152	11	79	K.AAQEALYVR.C	Fructose-bisphosphate aldolase-like protein	gi|7529717	*Arabidopsis thaliana*	At3g52930*
	11	79	K.GILAADESTGTIGK.R				At2g36460
121	p	26	R.NLLSVAYK.N	14-3-3-like protein GF14 Upsilon	gi|9755651	*Arabidopsis thaliana*	At5g16050*
	p	26	R.EENVYLAK.L				
	p	26	R.YEEMVEFMEK.V				
115	d, e	83, 80	R.GGGPTYLAIQSQPPGSVMGTLR.V	Bacterial-type phospho*enol*pyruvate carboxylase	gi|18073826	*Cycas revoluta*	At1g68750*
	d	83	K.FGLPQTAVR.Q				
99	o	35	R.LISQTISSLTTSLR.F	TUA1	gi|15217737	*Arabidopsis thaliana*	At1g64740*
	o	35	R.AVFLDLEPTVIDEVR.T				
81	m	43	K.ASNPFVNLK.K	Type IIIa membrane protein cp-wap13	gi|2218152	*Vigna unguiculata*	At3g02230*
	m	43	R.YVDAVLTIPK.G				
78	15	65	K.VGLAQMLR.G	Pyridoxine biosynthesis protein-like	gi|7320719	*Arabidopsis thaliana*	At2g38210*
	15	65	R.TKGEAGTGNIIEAVR.H				At2g38230
	15	65					At5g01410
53	f	72	R.IDAVDASTVK.R	Metalloendopeptidase	gi|15232845	*Arabidopsis thaliana*	At3g02090*
53	12	79	R.SQGSQGSGDGQGSVEGQQTPR.F	Zinc finger (C3HC4-type RING finger) family protein	gi|15231003	*Arabidopsis thaliana*	At3g19950*
43	3, 12	105, 79	K.LELSDIAGR.V	APUM5 (ARABIDOPSIS PUMILIO 5); RNA binding	gi|30685690	*Arabidopsis thaliana*	At3g20250*
41	e	80	R.MGIIRGIGR.G	T23J18.8	gi|6554181	*Arabidopsis thaliana*	At1g11410*
				S-locus protein kinase, putative	gi|15220362	*Arabidopsis thaliana*	At1g11340*

*; checked by RT-PCR (see Figure 1)

†; see Additional file 3
