# Supplementary material for: Characterization of bacterial-type phosphoenolpyruvate carboxylase expressed in male gametophyte of higher plants
Source: BMC Plant Biol. 2010 Sep 14;10:200. doi: 10.1186/1471-2229-10-200 (PMC2956549; doi:10.1186/1471-2229-10-200)
Supplement: Additional file 5 — Primers used in this study. Primers used in this study. [file 1471-2229-10-200-S5.PDF]

Primer name	Sequence (5'-3')	Application
ppc4-SALK-For	CGCCATAATTTACTGCGTTGACCAC	genotyping of *Atppc4* mutant line
LBc	TCAGGGCCAGGCGGTGAAG	genotyping of A*tppc4* mutant line
AT1G78900-F	GGAGGAAAGCTTACGACCTT	RT-PCR of lily ortholog
AT1G78900-R	GGCAACGAGGTGATTCATCA	RT-PCR of lily ortholog
AT2G07698-F	CGCCCAACTGGTATCGTTAT	RT-PCR of lily ortholog
AT2G07698-R	GCACGGGATGTGTAAGTTGC	RT-PCR of lily ortholog
AT1G53310-F	CAGGATTTGCACGGTGAAGA	RT-PCR of lily ortholog
AT1G53310-R	GCATATAGTTGAGCCAGACAG	RT-PCR of lily ortholog
AT3G52930-F	CAAGCAAATTCGCCGATGAG	RT-PCR of lily ortholog, also anneal to At2g36460
AT3G52930-R	GTAAGCGTTCTCATGAATGG	RT-PCR of lily ortholog, also anneal to At2g36460
AT5G16050-F	GATGTCTTCTGATTCGTCCC	RT-PCR of lily ortholog
AT5G16050-R	GCAAGAGCAATATCCTGAGC	RT-PCR of lily ortholog
AT1G68750-F	CGCGAAACGCTCATTGAAGA	RT-PCR of lily ortholog
AT1G68750-R	GAGCTCTAGCTGGAAGTTGT	RT-PCR of lily ortholog
AT1G64740-F	CCTTCTTCCTCGCGAAAACA	RT-PCR of lily ortholog
AT1G64740-R	CCCCCAACAGCATTGAATAC	RT-PCR of lily ortholog
AT3G2230-F	GGTGAACCACATCCCATTGT	RT-PCR of lily ortholog
AT3G2230-R	GTATCCACGGACGAAGTCAG	RT-PCR of lily ortholog
AT2G38210-F	CTATTCGGTTACTTAGAAGC	RT-PCR of lily ortholog, also anneal to At2g38230
AT2G38210-R	CTTAGCCCTCTTCACCGGAT	RT-PCR of lily ortholog, also anneal to At2g38230
AT3G02090-F	GAGGCGTTTATTCCTCACTC	RT-PCR of lily ortholog
AT3G02090-R	TGTATACGCGTTCAGGTGAC	RT-PCR of lily ortholog
AT3G19950-F	CCGGAGGTTGACAAGATGTT	RT-PCR of lily ortholog
AT3G19950-R	CCGAAGGATGATTCTCGATC	RT-PCR of lily ortholog
AT3G20250-F	CTTGAATCCGAGACTCCCT	RT-PCR of lily ortholog
AT3G20250-R	CTCGCGTTCAATCTCTCTGT	RT-PCR of lily ortholog
AT1G11410-F	CGAGCCTAAGACACCTCGAG	RT-PCR of lily ortholog
AT1G11410-R	CCTCAAGCTCTTCAAGTATG	RT-PCR of lily ortholog
AT1G11340-F	GGGGCGTGACTCACAGTTAA	RT-PCR of lily ortholog
AT1G11340-R	GGTTCCAACATACTATCTG	RT-PCR of lily ortholog
AT4G05050-F	ATGCAGATTTTCGTTAAGACTCTC	RT-PCR of lily ortholog
AT4G05050-R	CTAACCACCACGGAGCCTGAGAAC	RT-PCR of lily ortholog
PPC4-dTOPO-For	CACCATGACGGACACAACAGACGATATCGC	RT-PCR analysis of *Atppc4*
PPC4-dTOPO-Rev	TTAACCGGTATTTCTCATTCCTGCAGCAAT	RT-PCR analysis of *Atppc4*
AtEF1α-F	CATCAAGAACATGATCACTGGT	RT-PCR analysis of *Atppc4*
AtEF1α-R	GTCCCACTGGCACCGTTCCAAT	RT-PCR analysis of *Atppc4*
PPC1-3AB-F	GGAATTCCATATGGCGAATCGGAAGTTAG	anti-AtPTPC antibody production
PPC1-3AB-R	CGGGATCCTTAGGTGACTTCAGGTGTAACC	anti-AtPTPC antibody production
PPC4AB-F	GGAATTCCATATGACGGACACAACAGACG	anti-AtBTPC antibody production
PPC4AB-R	CGGGATCCTTAATCAACCTCTCTTATGTAC	anti-AtBTPC antibody production
PPC1-pro-F	CACCGATGATGGTCGGTTAAATACTTAAGT	cloning of *Atppc1* promoter
PPC1-pro-R	TTTTTCACCTGCTTCGCTCTGAAGTAAGAC	cloning of *Atppc1* promoter
PPC2-pro-F	CACCGATAAGAGACCGTAATAGACGTGGTA	cloning of *Atppc2* promoter
PPC2-pro-R	GGTTTGGTTGATGCTTTTTCCTCTCTCTCT	cloning of *Atppc2* promoter
PPC3-pro-F	CACCGTCGACGGCCACCACATCACTTTTGT	cloning of *Atppc3* promoter
PPC3-pro-R	GTCGACTATCGCCGATCAATCAAATCTTCT	cloning of *Atppc3* promoter
ppc4-promoterF	CTTCTCCTGTCGACATTATTATCGAACAC	cloning of *Atppc4* promoter
ppc4-promoterR	CGGGATCCTGTATCAATGAACAAGGTGAAG	cloning of *Atppc4* promoter
PPC4-1F	CGGGATCCATGACGGACACAACAGACGATA	cloning of genomic *Atppc4*
PPC4-3UTR-R	CATAAGAATGCGGCCGCGGACCCAAGAATGCATGTTTATCTCACTAT	cloning of genomic *Atppc4*
VenusF2	GGAGGTGGAGGTGGAGCTATGGTGAGCAAGGGCGAGGA	cloning of *Venus*
VenusR2	CCCAGCGGCCGCAGCAGCACCAGCGGCGGCGGTCACGAACTCCAGCA	cloning of *Venus*
